# Assessing the association between food environment and dietary inflammation by community type: a cross-sectional REGARDS study

**DOI:** 10.1186/s12942-023-00345-4

**Published:** 2023-09-20

**Authors:** Yasemin Algur, Pasquale E. Rummo, Tara P. McAlexander, S. Shanika A. De Silva, Gina S. Lovasi, Suzanne E. Judd, Victoria Ryan, Gargya Malla, Alain K. Koyama, David C. Lee, Lorna E. Thorpe, Leslie A. McClure

**Affiliations:** 1 Department of Epidemiology and Biostatistics, Drexel University Dornsife School of Public Health, Nesbitt Hall, 3215 Market Street, Philadelphia, PA 19104 USA; 2 Department of Population Health, New York University Grossman School of Medicine, New York, NY USA; 3 Department of Biostatistics, The University of Alabama at Birmingham School of Public Health, Birmingham, AL USA; 4 Department of Epidemiology, The University of Alabama at Birmingham School of Public Health, Birmingham, AL USA; 5 Division of Diabetes Translation, Centers for Disease Control and Prevention, Atlanta, GA USA; 6 Department of Emergency Medicine, New York University Grossman School of Medicine, New York, NY USA

**Keywords:** Diet, Restaurants, Supermarkets, Inflammation, Neighborhood characteristics, Census Tract, Surveys and questionnaires

## Abstract

**Background:**

Communities in the United States (US) exist on a continuum of urbanicity, which may inform how individuals interact with their food environment, and thus modify the relationship between food access and dietary behaviors.

**Objective:**

This cross-sectional study aims to examine the modifying effect of community type in the association between the relative availability of food outlets and dietary inflammation across the US.

**Methods:**

Using baseline data from the REasons for Geographic and Racial Differences in Stroke study (2003–2007), we calculated participants’ dietary inflammation score (DIS). Higher DIS indicates greater pro-inflammatory exposure. We defined our exposures as the relative availability of supermarkets and fast-food restaurants (percentage of food outlet type out of all food stores or restaurants, respectively) using street-network buffers around the population-weighted centroid of each participant’s census tract. We used 1-, 2-, 6-, and 10-mile (~ 2-, 3-, 10-, and 16 km) buffer sizes for higher density urban, lower density urban, suburban/small town, and rural community types, respectively. Using generalized estimating equations, we estimated the association between relative food outlet availability and DIS, controlling for individual and neighborhood socio-demographics and total food outlets. The percentage of supermarkets and fast-food restaurants were modeled together.

**Results:**

Participants (n = 20,322) were distributed across all community types: higher density urban (16.7%), lower density urban (39.8%), suburban/small town (19.3%), and rural (24.2%). Across all community types, mean DIS was − 0.004 (SD = 2.5; min = − 14.2, max = 9.9). DIS was associated with relative availability of fast-food restaurants, but not supermarkets. Association between fast-food restaurants and DIS varied by community type (*P* for interaction = 0.02). Increases in the relative availability of fast-food restaurants were associated with higher DIS in suburban/small towns and lower density urban areas (p-values < 0.01**)**; no significant associations were present in higher density urban or rural areas.

**Conclusions:**

The relative availability of fast-food restaurants was associated with higher DIS among participants residing in suburban/small town and lower density urban community types, suggesting that these communities might benefit most from interventions and policies that either promote restaurant diversity or expand healthier food options.

**Supplementary Information:**

The online version contains supplementary material available at 10.1186/s12942-023-00345-4.

## Background

Dietary risk factors are major contributors to non-communicable disease morbidity and mortality. In 2017, approximately 11 million deaths were attributable to dietary risk factors, primarily through cardiovascular disease, cancer, and diabetes [1]. According to the results from the 2017–2018 National Health and Nutrition Examination Survey (NHANES), an estimated 42.5% of U.S. adults aged 20 and over have obesity [2]. In 2016, excess weight was estimated to contribute to over 1300 deaths per day (nearly 500,000 per year) in the United States (US), surpassing the impact of smoking on excess mortality [3]. Additionally, obesity incurs a cost of nearly $173 billion per year on the US healthcare system [4]. Since the late 1970s, obesity prevalence has increased in all major US population subgroups [5–8], in part due to increased availability and affordability of energy dense foods [9] and subsequent changes in dietary patterns [10]. Interventions narrowly focused on the individual have had limited success, shifting public health thinking from a behavior-change perspective to an ecological approach [11–13]. With increased recognition of contextual factors as influencers of behavior and health, there is growing interest in interventions and policies aiming to modify the local food environment to improve dietary quality and associated health outcomes [14].

Differences in dietary quality have been reported across socioeconomic and racial subgroups [15]. Disparities in food access may partially explain these disparities in dietary quality [16, 17]. Studies have examined effect modification in the relationship between food environment and diet by individual-level factors such as race/ethnicity, income, and sex [18–22], with fewer studies incorporating neighborhood-level modifiers. Previous research has indicated that lower income and predominantly minority neighborhoods may have less access to healthy foods [23–25]. While few studies have attempted to distinguish between the influence of neighborhood income and racial composition on these disparities, one study reported that race disparities in supermarket availability persisted even after accounting for neighborhood income [25]. Moreover, research suggests that race and income disparities in supermarket availability may vary by community type. Specifically, these disparities may be more pronounced in low-density urban/suburban areas compared to high-density urban areas [26]. In addition, there may be differences in dietary outcomes across community types. Several studies, for example, have reported that rural residents consume fewer fruits and vegetables and more sweetened beverages compared to non-rural residents [27–29].

Communities in the US exist on an urban–rural spectrum, with varying interrelated built and social environment features that promote or hinder healthy living [30, 31]. These features tend to have differential co-occurrence patterns, exposing individuals to unique combinations of neighborhood features based on the community type they live in [30, 32–35]. Urban areas may have greater availability and accessibility of both healthy and less healthy food sources [36–40], healthier restaurant nutrition environments [38], and greater street connectivity, land use mix, and public transport use [30, 41, 42] compared to non-urban areas. Conversely, residents of non-urban spaces may spend more time in the car [6], travel further for food sources [43], have fewer supermarkets and greater availability of convenience stores [25, 44, 45], and have fewer opportunities for physical activity [27]. Finally, suburban grocery stores may have healthier in-store alternatives compared to urban stores, with fewer differences between suburban and rural stores [39].

Research on the link between the local food environment and diet is mixed [16, 24, 46–49], making targeted policy recommendations difficult [50]. One contributing factor to these differences may be the substantial variability in the definition and method used to operationalize food access [46–48]. A 2012 systematic review found that out of 20 studies that used GIS-based methods, 13 showed a significant association between spatial food availability and dietary outcomes [46]. Studies generally defined exposure by store type (e.g., access to supermarkets and/or fast-food restaurants) and most commonly assessed fruit and vegetable intake as the outcome variable. Studies of supermarket access occasionally reported a positive yet weak association with fruit and vegetable intake, while no significant associations were reported in other studies [19, 20, 51–54]. Several studies reported a link between fast food access and consumption, but others found null associations [55–62].

For dietary outcomes, some studies used customized screeners to assess specific food groups (e.g., fruits and vegetables), while others used validated semi-quantitative Food Frequency Questionnaires (FFQs) to evaluate dietary quality, derived from either USDA guidelines or principal components analysis [46]. Few studies have used a novel third approach, weighting aggregated foods groups derived from a FFQ by their strength of association with biomarkers of systemic inflammation. One such measure, which is used in the current study, is the Dietary Inflammation Score (DIS). While diet quality measures such as the Healthy Eating Index (HEI) and the Alternate Healthy Eating Index (AHEI) were designed to assess Americans’ adherence to US dietary recommendations, they were not designed to relate to the inflammatory mechanisms associated with chronic diseases such as diabetes. Dietary scores such as the DIS [65] have shown a stronger association with disease than the HEI [66–69]. In the Reasons for Geographic and Racial Differences in Stroke (REGARDS) sample, the DIS was associated with all-cause, all-cancer, and all-cardiovascular disease (CVD) mortality [70, 71], while markers of circulating inflammation have been associated with risks of CVD and type 2 diabetes in other large, prospective studies [72–74].

Limited research has explored the relationship between food environment and diet across diverse community types on a large geographic scale in the US [46, 47]. To our knowledge, no prior studies have examined how community type may influence this association in urban, suburban, and rural environments in the US. In a recent study, we observed a stronger association between the relative availability of fast-food restaurants and dietary inflammation while utilizing community type-specific buffer-based measures compared to uniform distances [75]. These results support the hypothesis that the relationship between the food environment and diet varies across different community types [76]. The objective of our current study is to determine whether there are community-type differences in the association between geographic availability of food outlets and pro-inflammatory diet among participants enrolled in the REGARDS study between 2003 and 2007.

## Methods

### Study sample and procedures

The REGARDS study is a population-based cohort of 30,239 non-Hispanic Black adults and non-Hispanic White adults aged ≥ 45 years, enrolled between 2003 and 2007 from the contiguous US, with oversampling of Black individuals (42%) and residents of the Stroke Belt (56%) [83]. The Stroke Belt refers to a region of high stroke mortality in the Southeastern US and is commonly defined as including eight southern states (North Carolina, South Carolina, Georgia, Tennessee, Mississippi, Alabama, Louisiana, and Arkansas). The goals of the REGARDS study were to examine regional and racial differences in stroke mortality and cognitive functioning. As a population-based prospective cohort followed for over 20 years, the REGARDS study participants present an important target population due to the study’s expansive geographic coverage and oversampling of racial and geographic subgroups that continue to be at higher risk for chronic diseases. Study objectives, design and methods are described in detail elsewhere [83]. Briefly, participants were identified using stratified random sampling and were recruited by mail and then contacted by phone, during which time baseline (2003–2007) demographic and medical history data were collected using computer-assisted telephone interviews. An in-home physical assessment followed, during which blood and urine samples were collected, as well as several anthropometric traits (e.g., height and weight) and blood pressure measured. Participants were left a packet of self-administered forms to complete and return to the Coordinating Center at the University of Alabama at Birmingham, including the Block 98 Semi-Quantitative Food Frequency Questionnaire (FFQ), used to measure dietary intake [83–85]. All participants provided written informed consent, and Institutional Review Board approval was obtained by all participating institutions.

### Exposures

#### Food environment

Establishment data from the Retail Environment and Cardiovascular Disease (RECVD) study [86] were used to define food outlet availability. The RECVD study licensed the National Establishment Time Series (NETS) Database from Walls & Associates (Walls & Associates, Denver, CO), who prepared annual establishment information collected by Dun and Bradstreet (D&B, Short Hills, NJ). The RECVD team re-geocoded the NETS data to improve locational accuracy and assigned establishments to subcategories using Standard Industrial Classification (SIC) codes, employee and sales information, and chain names obtained from Technomic/Restaurants and Institutions (R&I) and TDLinx. Details on classification methods are described elsewhere [87]. Briefly, the RECVD team improved the initial classification of the NETS data, which relied on SIC codes, by conducting systematic checks and refining it further through name searches to establish a final classification. We linked these data, reflecting a snapshot of food establishments open in January of a given year, to the year of cohort entry (baseline) in the REGARDS sample. Individual-level data, including demographics and dietary information, were collected during this time.

Food environment measures were constructed and operationalized through the work by the Diabetes Location, Environmental Attributes, and Disparities (LEAD) Network [77], a CDC-funded research collaboration among Drexel University, Geisinger-Johns Hopkins University, New York University Grossman School of Medicine, and University of Alabama at Birmingham. For our analyses, we used measures of relative availability of two food outlet types: the percentage of supermarkets out of all food stores, and the percentage of fast-food restaurants out of all restaurants, similar to other work by the LEAD Network [75, 78–80]. We used relative measures of food environment rather than absolute measures, as they have been shown to predict dietary behavior more consistently [20, 75, 76, 88]. Absolute measures focus on quantifying the food environment without accounting for the influence of other food retailers, such as the number of supermarkets in a given area [89]. On the other hand, relative measures take into account the presence of other food retailers that may influence dietary choices [20]. This indicates that considering the full range of options may be more important in capturing foodscape exposure.

We chose not to collapse across different food outlet categories to define healthy or unhealthy food outlets since many food stores sell both healthy and unhealthy food items and there is no clear approach for determining the healthfulness of food establishments. However, supermarkets are generally considered to offer a mix of healthy and unhealthy food options [63] while foods consumed away from home at fast-food restaurants is generally of lower dietary quality [64]. Briefly, the supermarkets category included three mutually exclusive subcategories: supermarkets, supercenters, and medium-sized grocers. Medium-sized grocers were defined as stores offering more grocery options than convenience stores and small grocers/bodegas but fewer than supermarkets. The “all food stores” category included food stores primarily consumed off-premises, including wholesale/warehouse club stores, convenience stores, and small grocers/bodegas. Fast food restaurants were defined as quick-service restaurants specializing in low preparation time foods that are eaten cafeteria-style (no waiter service) or takeaway.

Following expert consensus by the Diabetes LEAD Network, we operationalized our measures using a street-network buffer with distances of 1, 2, 6, and 10 miles (1.6, 3.2, 9.7, and 16.1 km, rounded to the nearest tenth) from the population-weighted centroid of the census tract where REGARDS participants lived at baseline. Henceforth, we will refer to buffer distances in kilometers, rounded to the nearest whole number (2, 3, 10, 16 km). In our previous REGARDS work [75], we compared geospatial measures of the food environment across the US and found similar effect estimates for DIS between administrative (population-weighted centroid) and egocentric (residential address) network buffers. For the current study analyzing this relationship by community type, we used the administrative buffer, which has advantages over person-based buffers in studies where obtaining exact participant addresses may not be feasible due to privacy concerns or wide geographic coverage. The network buffer was created using the “generalized” polygon option and default settings in ArcGIS Pro 2.4.2. Street network data were obtained from ESRI’s ArcGIS StreetMap Premium 2019 release. The buffer distances were chosen based on the work by the Diabetes LEAD Network using the National Household Food Acquisition and Purchase Survey (FoodAPS) [90], which includes data on driving distances between household residence and primary food store and indicators for rural and nonmetropolitan residence for survey participants.

#### Community type

All Network analyses were stratified by a four-level community type classification to account for non-overlapping distributions of community factors across different communities. The four-level improved community type classification was developed by the LEAD Network as a modification of the United States Department of Agriculture’s Rural–Urban Commuting Area (RUCA) codes at the census tract level. Details on its construction, including a comparison with existing classifications, is described elsewhere [30]. Briefly, compared to RUCA classifications used in three other studies, the LEAD community type classification demonstrated greater variability in distributions of land characteristics, such as street connectivity and percent developed land, of census tracts across community types. Moreover, our community type classification provided a more granular delineation of census tracts within urban areas, resulting in two distinct urban categories based on land area (i.e., “higher density urban” and “lower density urban”). Similar to previous work by the LEAD Network [75, 78–80], we used buffer-based measures of the food environment tailored to the community type of participants’ residential census tract. Following the Network’s approach, we chose different buffer sizes a priori to define our food environment measures by the community type of participants’ residential census tract. Namely, the 2-, 3-, 10-, and 16 km buffer distances were assigned to participants residing in higher density urban, lower density urban, suburban/small town, and rural census tracts, respectively, to provide buffers appropriately scaled to each community type. The 2 km network buffer measures were calculated using walking distance, and other distances were calculated using driving distance.

#### Covariates

Individual-level covariates were age (continuous), sex (female or male), education (less than high school, high school graduate, some college, college graduate and above), race (non-Hispanic Black persons, non-Hispanic White persons), and annual household income (< $20,000, $20,000 to $34,000, $35,000 to $74,000, ≥ $75,000, and refused). Neighborhood-level covariates were neighborhood socioeconomic environment (NSEE), community type classification, and density of total food outlets. NSEE was developed and operationalized by the LEAD Network as a z-score sum of six census variables, scaled to 0–100 range, based on previous work [91]. The six indicators of NSEE were percentage of males and females with less than a high school education, percentage of males and females unemployed, percentage of households earning less than $30,000 per year, percentage of population with income below poverty level, percentage of households on public assistance, and percentage of occupied housing units with no vehicle. Higher NSEE scores indicate more socioeconomic disadvantage. Participants were classified by the location of their residential census tracts into one of four LEAD community types: higher density urban, lower density urban, suburban/small town, and rural [30]. We defined the density of total food outlets (continuous) as the sum of all food stores and all restaurants/eating places per km^2^.

### Outcomes

The Block-98 FFQ assessed usual quantity and frequency of 109 food items consumed over the previous year. Frequency was assessed by asking how often, on average, participants consumed the food, with nine response options ranging from “never” to “every day”. To assess food quantity, participants were prompted to refer to pictures for standard food portion sizes. Frequency and quantity were multiplied to determine daily intake (g/day) for each item by NutritionQuest (Berkeley, CA). Our primary outcome was the dietary inflammation score (DIS) [92], a validated measure of exposure to a pro-inflammatory diet. DIS was associated with incident type 2 diabetes and mortality risk [71, 93]. Items on the FFQ were aggregated to 19 food groups selected a priori and weighted based on their strength of associations with an inflammatory biomarker score, representing systemic inflammation. Examples of proinflammatory food groups include processed meats, added sugars, and refined grains. The weighted components were summed to generate a composite score. Higher scores indicate more proinflammatory (relative to anti-inflammatory) diets (theoretical range: − 14.9–12.8).

We included the Mediterranean diet score as a secondary outcome. Greater adherence to a traditional Mediterranean diet has been linked with lower mortality and reduced risk of chronic disease in various populations [94], including REGARDS participants [95–97]. Details on methods for its construction were published previously [98]. In short, nine food groups were selected from items on the FFQ and were each assigned a score of 0 or 1 based on a comparison of dietary intake with thresholds for a given category. For example, a point was added if consumption was above the sex-specific median for food groups designated as “beneficial” or was below the median for those designated as “detrimental”. Scores for the nine food groups were summed, resulting in a theoretical range of 0 to 9, with a higher score reflecting higher adherence to a Mediterranean diet.

### Statistical analysis

We compared characteristics of the REGARDS sample across community types using χ^2^ tests for categorical variables and ANOVA for continuous variables. We summarized participants’ food environment measures by community type designation. To examine the relationship between food environment measures and DIS, we used generalized estimating equations (GEEs) with an identity link, exchangeable correlation structure, and robust standard errors, accounting for clustering at the census tract level. Supermarkets and fast-food restaurants were modeled together to examine their independent associations with diet. Using buffer distances tailored to community type, we assessed interactions by community type using a cross-product term, with rural as our reference group (i.e., percentage of supermarkets or fast-food restaurants x community type) and conducted stratified analyses by community type. We tested simple slopes of food environment measures at different categories of community type. As a sensitivity analysis, we examined regression coefficients of non-primary buffer sizes for each community type in analyses stratified by community type. In all models, we controlled for individual-level covariates (age, sex, education, race, household income) and NSEE, all of which are plausibility associated with our exposure and outcomes [99, 100]. We also controlled for total food outlets to address a potential limitation of percentage measures not reflecting the quantity of food outlets [101, 102]. We followed the same approach to examine the relationship between food environment measures and Mediterranean diet score (identity link). All analyses were performed in SAS version 9.4 (SAS Institute Inc., Cary, NC, USA) and RStudio [103]. All significance tests of our non-stratified models employed an unadjusted alpha level of *P* < 0.05. To account for the family-wise error rate (FWER) in our subgroup analyses, we’ve calculated a Bonferroni-adjusted alpha value of *P* ≤ 0.01 by diving our original alpha value by the number of tests performed (0.05/4) [104].

## Results

For this cross-sectional, secondary analysis, we excluded 56 participants due to anomalous data. Of the remaining 30,183, we excluded those missing dietary data (n = 9,643), census tract identifier (n = 287) or sociodemographic data (n = 9) at baseline, resulting in a sample size of 20,322 participants. Reasons why dietary data were missing include unreturned FFQ, incomplete FFQ, or implausible caloric intake [105]. Participants excluded due to missing data were more likely to be a Black person, male, and lower income, and have less than a high school education (Additional file 2: Table S1).

Approximately 16.7%, 39.8%, 19.3%, and 24.2% of participants resided in higher density urban, lower density urban, suburban/small town, and rural communities, respectively. Across all community types, the mean DIS and Mediterranean diet scores for the sample were − 0.004 (SD = 2.52) and 4.4 (SD = 1.7), respectively (Table 1). Participants residing in higher density urban areas had the highest mean DIS (0.17, SD = 2.64), indicating greater consumption of pro-inflammatory foods, while those in suburban/small towns had the lowest mean DIS (-0.14, SD = 2.50) (p < 0.0001). Participants’ mean Mediterranean diet scores were highest in higher density urban areas (4.48, SD = 1.72) and lowest in rural (4.15, SD = 1.65) (Table 1) (p < 0.0001), although differences were not necessarily large. Higher density urban areas had both the highest DIS and the highest Mediterranean diet score. A cross-tabulation of these variables indicated that it was mostly different individuals contributing to the high scores for each measure in higher density urban areas (Additional file 3: Table S2). Across all community types, the relative availability of fast-food restaurants was higher compared to supermarkets (Table 2). There were differences in the availability of food outlets across community types.

**Table 1:** Unadjusted sociodemographic characteristics by community type in a sample of older US adults

Characteristic	All	Higher density urban	Lower density urban	Suburban/Small town	Rural	p-value
	(n = 20,322)	(n = 3386)	(n = 8089)	(n = 3920)	(n = 4927)	
Age, mean (SD)^a^	64.82 (9.23)	64.89 (9.30)	65.30 (9.37)	64.75 (9.18)	64.06 (8.94)	<0 .0001
Sex, n (%)^a^						
Male	8977 (44.17)	1288 (38.04)	3632 (44.90)	1857 (47.37)	2200 (44.65)	<0 .0001
Female	11,345 (55.83)	2098 (61.96)	4457 (55.10)	2063 (52.63)	2727 (55.35)	
Education, n (%)^a^						
Less than high school	1923 (9.46)	388 (11.46)	655 (8.10)	327 (8.34)	553 (11.22)	<0 .0001
High school graduate	5158 (25.38)	833 (24.60)	1817 (22.46)	964 (24.59)	1544 (31.34)	
Some college	5571 (27.41)	974 (28.76)	2258 (27.91)	1049 (26.76)	1290 (26.18)	
College graduate and above	7670 (37.74)	1191 (35.16)	3359 (41.53)	1580 (40.31)	1540 (31.26)	
Race, n (%)^a^						
Black	6821 (33.56)	2190 (64.68)	3018 (37.31)	784 (20.00)	829 (16.83)	< 0.0001
White	13,501 (66.44)	1196 (35.32)	5071 (62.69)	3136 (80.00)	4098 (83.17)	
Income, n (%)^a^						
< $20,000	3159 (15.54)	685 (20.23)	1164 (14.39)	496 (12.65)	814 (16.52)	< 0.0001
$20,000−$34,000	4909 (24.16)	896 (26.46)	1876 (23.19)	852 (21.73)	1285 (26.08)	
$35,000−$74,000	6409 (31.52)	938 (27.70)	2632 (32.54)	1313 (33.49)	1523 (30.91)	
$75,000 and above	3533 (17.39)	450 (13.29)	1513 (18.70)	808 (20.61)	762 (15.47)	
Refused	2315 (11.39)	417 (12.32)	904 (11.18)	451 (11.51)	543 (11.02)	
NSEE, mean (SD)^a,b^	19.50 (11.18)	27.37 (13.03)	17.22 (11.06)	16.33 (10.08)	20.36 (7.59)	< 0.0001
DIS, mean (SD)^c^	− 0.004 (2.52)	0.17 (2.64)	–0.08 (2.52)	− 0.14 (2.50)	0.10 (2.45)	< 0.0001
Mediterranean Diet Score, mean (SD)^d^	4.36 (1.70)	4.48 (1.72)	4.45 (1.70)	4.33 (1.70)	4.15 (1.65)	< 0.0001

Statistical significance is *P* < 0.05 for the χ^2^ and ANOVA tests between REGARDS participants residing in higher density urban, lower density urban, suburban/small town, and rural communities; *SD* standard deviation

^a^n = 20,322. Participants aged ≥ 45 years were enrolled in the REGARDS study from 2003 to 2007. Sample excludes those with anomalous data (n = 56) and those missing DIS, census tract identifier or sociodemographic data (n = 9861)

^b^NSEE = Neighborhood social and economic environment is a z‐score sum of 6 US census‐derived variables, with sums scaled between 0 and 100. A higher NSEE score indicates more socioeconomic disadvantage

^c^Theoretical range: − 14.9–12.8. A higher score indicates greater exposure to pro-inflammatory foods

^d^Theoretical range: 0–9. A higher score indicates greater adherence to a Mediterranean diet

^d^n = 21,042. Sample excludes those with anomalous data (n = 56) and those missing Mediterranean Diet Score, census tract identifier or sociodemographic data (n = 9141)

**Table 2:** Relative measures of the food environment by community type

	Higher density urban	Lower density urban	Suburban/Small town	Rural	p-value
Supermarkets, mean (SD)^a^					
Percentage, tailored	0.10 (0.07)	0.11 (0.08)	0.12 (0.05)	0.13 (0.07)	<0 .0001
Fast food restaurants, mean (SD)^a^					
Percentage, tailored	0.27 (0.15)	0.34 (0.14)	0.35 (0.10)	0.32 (0.14)	< 0.0001
Total food outlets, mean (SD)^b^					
Density, tailored	14.37 (18.39)	5.03 (3.12)	1.64 (1.21)	0.30 (0.30)	<0 .0001

Percentages were expressed as decimals. Summary table reflects 5 year averages of food outlets from 2003 to 2007. We assigned 2-, 3-, 10-, and 16-km (1-, 2-, 6-, and 10-mile) buffer distances to participants residing in higher density urban, lower density urban, suburban/small town, and rural census tracts, respectively. Buffer sizes are represented in kilometers rounded to the nearest whole number

^a^All food stores and all restaurants were used as the denominators for the percentage of supermarkets and fast-food restaurants, respectively

^b^Total food outlets was included as a covariate in all regression models

**Fig. 1:**
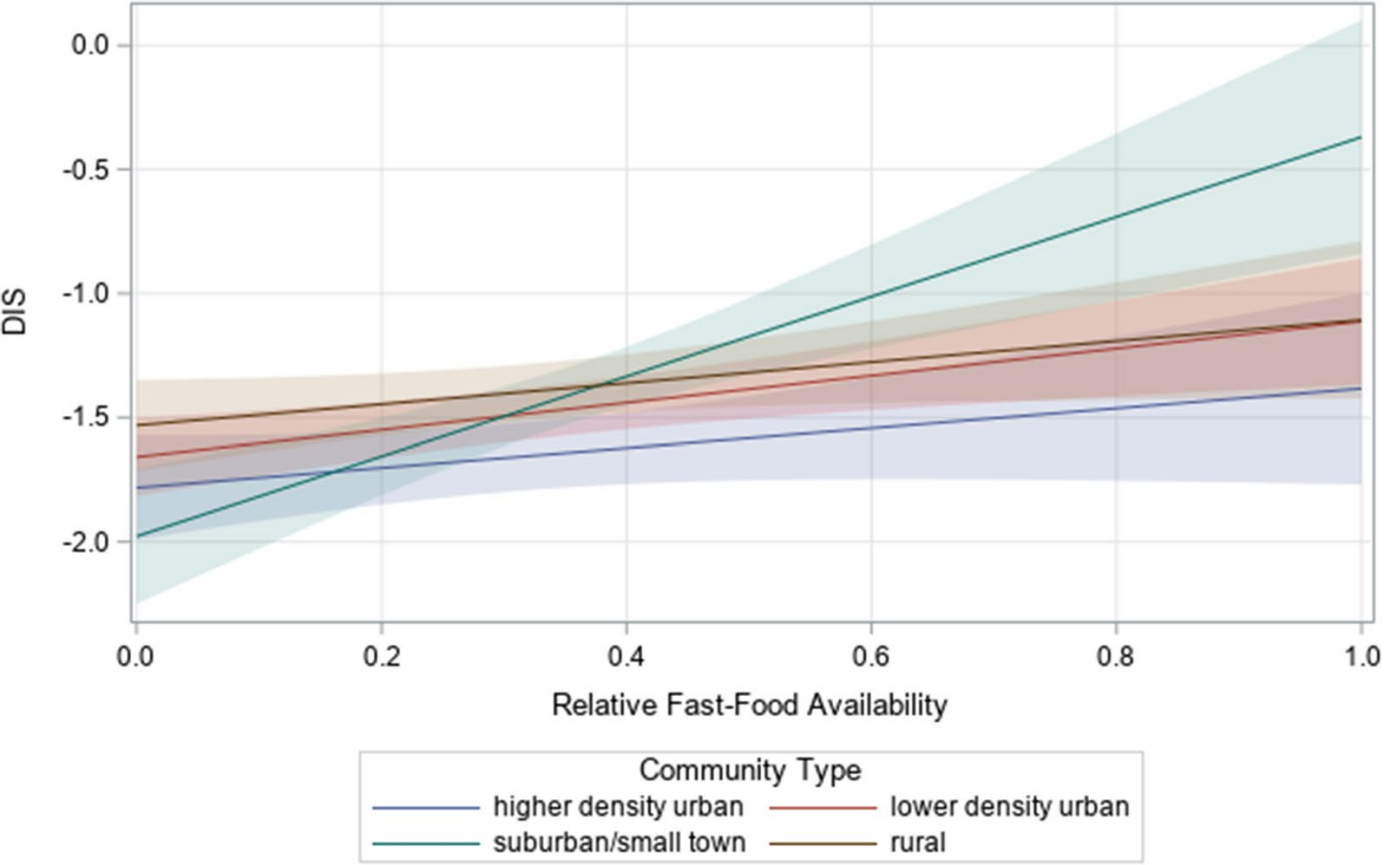
Plotting simple slopes of fast-food restaurant availability with 95% confidence intervals by community type. Percentages were expressed as decimals

Using buffer sizes tailored to community type, we found a significant association between the relative availability of fast-food restaurants and DIS (*β* = 0.59, SE = 0.12, *P* < 0.001), and this association varied by community type (*P* for interaction = 0.02) (Table 3). Increases in the relative availability of fast-food restaurants were associated with higher DIS (indicating greater exposure to pro-inflammatory foods) in lower density urban (*β* = 0.55, SE = 0.18, *P* < 0.01) and suburban/small town (*β* = 1.50, SE = 0.37, *P* < 0.001) areas (Table 4). No significant associations were found in other community types. Participants residing in suburban/small towns had significantly higher DIS associated with increases in the availability of fast-food restaurants compared to participants residing in other community types (*P* values < 0.01) (Fig. 1). No significant associations were found between the relative availability of supermarkets and DIS.

**Table 3:** Model-based associations of the food environment with dietary inflammation score (DIS)

	Main effects model		Interaction model	
	β (SE)	p-value	β (SE)	p-value
Supermarkets, tailored^a^	0.33 (0.22)	0.13	0.34 (0.22)	0.13
Fast-food restaurants, tailored^a^	**0.59 (0.12)**	** <0 .001**	0.42 (0.23)	0.06
Community type				
Higher density urban	− **0.26 (0.07)**	** < 0.001**	0.35 (-0.71)	0.94
Lower density urban	− 0.09 (0.05)	0.06	0.29 (− 0.44)	0.67
Suburban/small town	− 0.04 (0.05)	0.45	**0.42 (0.35)**	**0.01**
Rural (reference)				
Fast-food restaurants X Community type^b^				
Higher density urban			− 0.03 (0.35)	0.94
Lower density urban			0.12 (0.29)	0.67
Suburban/small town			**1.18 (0.42)**	**0.01**
Rural (reference)				

Bold denotes statistically significant at α < 0.05 level. Supermarkets and fast-food restaurants were modeled together. We controlled for individual-level covariates, NSEE, and total food outlets in all models. Higher DIS scores indicate more proinflammatory diets (theoretical range: − 14.9–12.8)

^a^We tailored buffer sizes to each community type using 2-, 3-, 10-, and 16 km (1-, 2-, 6-, and 10-mile) buffers for higher density urban, lower density urban, suburban/small town, and rural areas, respectively. Buffer sizes are represented in kilometers rounded to the nearest whole number

^b^P-value for overall test of interaction = 0.02

**Table 4:** Model-based associations of the food environment with DIS stratified by community type (n = 20,322)

	Higher Density Urban		Lower Density Urban		Suburban/small town		Rural	
	β (SE)	p-value	β (SE)	p-value	β (SE)	p-value	β (SE)	p-value
Supermarkets								
Percentage, tailored^a^	0.67 (0.54)	0.21	0.36 (0.32)	0.27	− 0.16 (0.66)	0.81	− 0.03 (0.43)	0.94
Fast-food restaurants								
Percentage, tailored^a^	0.46 (0.27)	0.09	**0.55 (0.18)**	** < 0.01**	**1.50 (0.37)**	** < .001**	0.42 (0.23)	0.06

Bold denotes statistically significant at Bonferroni-corrected α < 0.01 level. Supermarkets and fast-food restaurants were modeled together. We controlled for individual-level covariates, NSEE, and total food outlets in all models. Higher scores indicate more proinflammatory diets (theoretical range: − 14.9–12.8)

^a^We tailored buffer sizes to each community type using 2-, 3-, 10-, and 16 km (1-, 2-, 6-, and 10-mile) buffers for higher density urban, lower density urban, suburban/small town, and rural areas, respectively. Buffer sizes are represented in kilometers rounded to the nearest whole number

In secondary analyses, the associations between the relative availability of food outlets and Mediterranean diet score were not significant (Additional file 4: Table S3). In stratified analyses by community type, we observed an association between the percentage of supermarkets and Mediterranean diet score in higher density urban areas (Additional file 5: Table S4). No significant associations were observed between the fast-food measures and Mediterranean diet score in any of the community types.

**Fig. 2:**
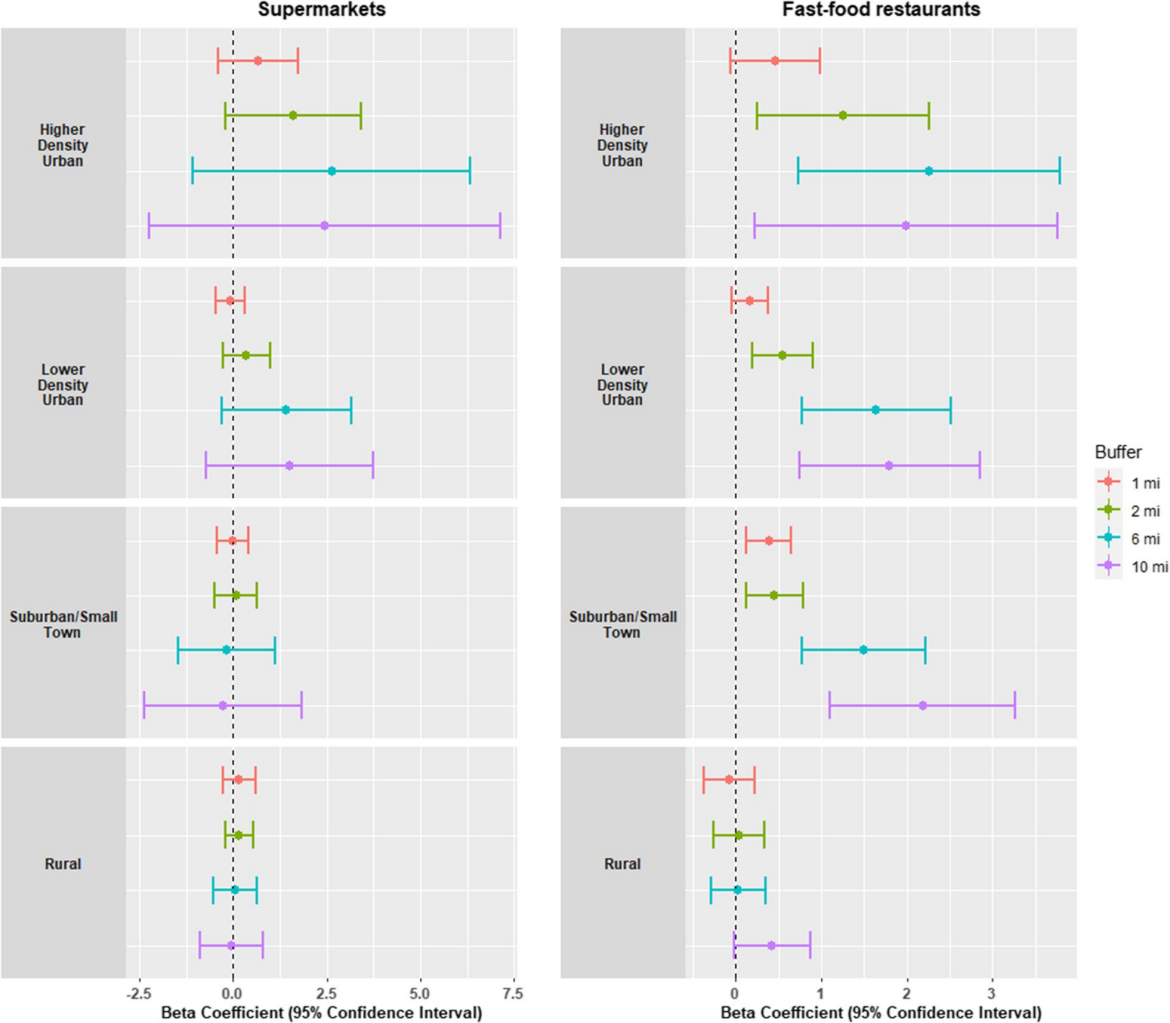
Estimates for the association between food access and DIS by buffer size and community type. Figure shows increase in DIS per each 1-unit increase in supermarket / fast-food restaurant availability. Food access is defined as the relative availability of two food outlets: the percentage of supermarkets out of all food stores, and the percentage of fast-food restaurants out of all restaurants

In sensitivity analyses, the relative availability of supermarkets and fast-food restaurants increased as buffer size increased (Additional file 6: Table S5). Buffer size influenced associations between food environment and DIS, but not Mediterranean diet score (Additional files 7, 8: Tables S7, S8). Using larger buffer sizes, increases in the availability of fast-food restaurants were associated with higher DIS for participants residing in higher density urban areas (p-values < 0.04), with overlapping confidence intervals between 10- and 16 km buffers (Fig. 2). No significant associations were found between fast-food restaurants and DIS in rural areas, nor between supermarkets and DIS in any of the community types regardless of buffer size. There was no significant variability in effect size across buffer size for Mediterranean diet score (Additional file 1: Figure S1).

## Discussion

In this large cohort of participants from across the contiguous US, we assessed whether relative availability of supermarkets and fast-food restaurants were associated with DIS and Mediterranean diet score. We found that there was an association between relative availability of fast-food restaurants and DIS, and that this association varied by community type. No associations were observed between supermarkets and DIS, and either fast-food restaurants or supermarkets and Mediterranean diet score.

Our previous paper demonstrated the utility of tailoring empirically derived buffer measures to community type [75], and our current analyses support examining associations between food environment and diet using tailored buffer measures stratified by community type, similar to previous work by the LEAD Network with type 2 diabetes as the outcome [78–82]. We observed the strongest association between relative fast-food availability and DIS in suburban/small towns compared to other community types, with buffer sizes selected a priori for each community type. This may be important given that a one-point increase in DIS was associated with a 2–3% higher risk of all-cause mortality in a prospective cohort study of women [70]. In a study in Denmark, increasing distance to nearest fast-food outlet was associated with increased odds of frequent fast-food intake among car owners living in suburban municipalities, while the opposite association was found among residents of urban municipalities [61]. In suburban/small towns, car dependency and lack of walkability may inform behaviors related to fast-food restaurant visits. In our study, participants residing in suburban/small towns had higher relative availability of fast-food restaurants compared to other community types. While previous research reported higher availability of fast-food restaurants in urban versus rural areas [40], consistent with other LEAD papers [78, 79], our study showed higher relative availability of fast-food restaurants in suburban/small towns compared to urban and rural areas.

Our results showed a significant association between relative fast-food availability and DIS in lower density urban areas. In contrast, a US study that differentiated between high- and low-density urbanicity did not find significant associations between neighborhood fast-food availability and consumption in either community type [58]. However, the study focused on chain restaurants only, potentially underestimating the full extent of fast-food exposure, and used a density measure with a uniform buffer size (3 km) across the different areas, without any further delineation of non-urban areas.

We did not find significant associations between fast-food restaurants and DIS in higher density urban or rural areas. In urban settings, fast-food restaurant availability may be less relevant to dietary inflammation compared to general food consumption patterns, such as a low proportion of meals consumed that are prepared at home. It may also be the case that urban residents can easily access healthier alternatives or utilize other food establishments for eating out of the home. However, in contrast to our findings, a study in metropolitan Melbourne, Australia [55] found a significant association between variety of fast-food restaurants and fast-food purchasing, but the study used a larger (3 km / ~ 1.9 mi) road network distance and measured fast food access differently compared to our study. In rural areas, it is possible that our definition for fast-food availability did not fully capture fast-food exposure. Convenience stores, and even wait service restaurants may offer more opportunities for procurement of fast food and have greater spatial accessibility than traditional fast-food restaurants in rural areas [44, 45]. However, a US study among rural adults found fast-food consumption was independently associated with both proximity and coverage of traditional and non-traditional fast-food outlets [62]. Finally, our lack of adjustment for vehicle ownership may have obscured associations in rural areas.

Our study findings did not support associations between supermarkets and diet quality scores, which contrasted with our original hypothesis. Studies of supermarket availability and accessibility have demonstrated mixed associations with dietary outcomes [49, 53], with relative measures (e.g., proportion) showing more consistency in associations with dietary behaviors compared to absolute measures (e.g., density) [20, 54, 76]. While mixed findings in the literature have been attributed to differences in measurement of both the exposure and outcome, it is likely that a purely spatial measure cannot capture all salient aspects of food access such as affordability, food choice, and acceptability/quality. Furthermore, it may be important to incorporate measures of both community and consumer food environments, accounting for in-store variation in healthy to unhealthy food items.

In sensitivity analyses, in contrast to our original hypothesis, we found that a larger buffer size (e.g., 10, 16 km) for fast-food restaurants had a larger impact on DIS regardless of community type, except for rural areas, where no significant associations were observed. While prior research informed our choice of buffer size for relevant food accessibility in different communities [43, 90], there may be factors other than proximity influencing food outlet choice.

As with all research, our study had some limitations. We excluded a third of participants from the parent REGARDS study who were missing dietary data, which may introduce bias. To partially address this issue, we accounted for personal characteristics that were associated with missingness in dietary data in our sample, which would have reduced bias if the data were missing at random (MAR). However, we were unable to implement an imputation method for missing individual FFQ items to account for data missing not at random (MNAR) because the majority of missingness in our sample was due to unreturned FFQ. Our study lacked information regarding in-store characteristics (e.g., variety of healthy and unhealthy foods at supermarkets), choice of food outlet, and individual food purchasing behaviors at food outlets. Our exposure area focused on residential neighborhoods and was static, preventing us from accounting for exposure in activity spaces. Specifically, a portion of food away from home may be consumed at eating places in or near the workplace. Depending on where the workplace is relative to the home, these establishments may or may not be captured by residential buffers, and the impact could differ by community type because of varying buffer size. While we have chosen buffer distances using prior research on food acquisition behaviors, we do not know whether they applied to our study participants. Our study was not designed to explore the impact of individual income on the food environment-diet relationship; however, future research should examine differences by household income in the association between food environment and diet stratified by community type. There is no universally accepted definition for community type, and our findings may be influenced by how we measured it. Future research should explore the impact of different definitions of community type on associations between food environment and diet. Finally, we could not account for vehicle ownership, which may have influenced our associations in non-urban areas. Neighborhood features acting as facilitators or barriers to food access may differ by community type, and future research could examine different moderators/mediators of the relationship between food environment and diet across community type. To further delineate differences by community type, it may be informative to compare objective measures of food access with measures of perceived food availability and accessibility.

Our study has several strengths. We used a tract-level community type measure that differentiates between four community types across the US, with buffer distances tailored to community type based on prior reporting of distance to primary food store in two national samples [43, 90]. Some studies have used survey-based measures of the perceived availability of food sources, but these measures may introduce same-source bias, or a spurious associations between self-reported neighborhood conditions and health outcomes [46]. For the current study,

we used an objective and reproducible method for measuring food access for a national sample using data that are available from external sources. We defined our exposures using administrative buffers (around population-weighted centroid of participants’ residential census tract), which have performed similarly to egocentric buffers [75] and offer a balanced approach for protecting confidentiality. We controlled for both neighborhood- and individual-level confounders (NSEE, household income), potentially reducing residual confounding in non-urban areas where neighborhood and individual-level measures may be less correlated [106]. Finally, our choice of health measures was guided by the Diabetes LEAD Network. The Network aims to identify modifiable community contributors to geographic disparities in type 2 diabetes risk across the US. To address this aim, the Network has defined and developed harmonized measures of various community domains, including food, physical activity, and neighborhood socioeconomic environments. Several LEAD Network papers have been published examining features of the built environment and their relationship with various health outcomes using diverse sample populations incorporating a variety of community types across the US [75, 78–82].

## Conclusion

In conclusion, we found an association between relative availability of fast-food restaurants and DIS, and this association varied by community type. Our findings support examining associations between food environment and diet using tailored buffer-based measures stratified by community type. Interventions could focus on restaurant diversity to mitigate dietary inflammation, especially in suburban/small town areas. Local governments could consider increased messaging around healthier food choices and offer potential incentives to restaurants providing foods that are less likely to cause inflammation. However, formal policy analysis should occur before policies are implemented.

### Supplementary Information

**Additional file 1: Figure S1.** Estimates for the association between food access and Mediterranean diet score by buffer size and community type.

**Additional file 2: Table S1.** Unadjusted sociodemographic characteristics of REGARDS participants excluded due to missing data.

**Additional file 3: Table S2.** Cross-tabulations of dietary scores across individuals living in higher density urban census tracts (n=3389).

**Additional file 4: Table S3.** Model-based associations of the food environment with Mediterranean diet score (n=20,322).

**Additional file 5: Table S4.** Model-based associations of the food environment with Mediterranean diet score stratified by community type (n=20,322).

**Additional file 6: Table S5.** Relative measures of the food environment by community type and buffer size.

**Additional file 7:** **Table S6.** Model-based associations of the food environment with DIS by buffer size and community type.

**Additional file 8: Table S7.** Model-based associations of the food environment with Mediterranean diet score by community type and buffer size.

## Data Availability

The following two datasets used in the current study are publicly available via Inter-university Consortium for Political and Social Research (ICPSR): US Census Tract Community Type Classification and Neighborhood Social and Economic Environment Score for 2000 and 2010, from the Diabetes Location, Environmental Attributes, and Disparities (LEAD) Network. Data Access Link: https://doi.org/10.3886/ICPSR38645.v1. Data on food environment cannot be shared publicly due to the terms and conditions of the licensed data. Data are available for researchers who meet the criteria to work with the licensed data. Contact gslovasiresearch@gmail.com or visit https://sites.google.com/view/recvd-team-project-site/home for more information.

## Abbreviations

Diabetes LEAD Network: Diabetes Location, Environmental Attributes, and Disparities Network
DIS: Dietary inflammation score
FFQ: Food frequency questionnaire
FoodAPS: National Household Food Acquisition and Purchase Survey
NETS: National Establishment Time Series
NSEE: Neighborhood socioeconomic environment
RECVD: Retail Environment and Cardiovascular Disease
REGARDS: Reasons for Geographic and Racial Differences in Stroke
