# Supplementary material for: Assessing the association between food environment and dietary inflammation by community type: a cross-sectional REGARDS study
Source: Int J Health Geogr. 2023 Sep 20;22:24. doi: 10.1186/s12942-023-00345-4 (PMC10510199; doi:10.1186/s12942-023-00345-4)
Supplement: Supplementary file 1 — Additional file 1: Figure S1. Estimates for the association between food access and Mediterranean diet score by buffer size and community type. [file 12942_2023_345_MOESM1_ESM.docx]

**SUPPLEMENTARY FIGURE 1. Estimates for the association between food access and Mediterranean diet score by buffer size and community type**


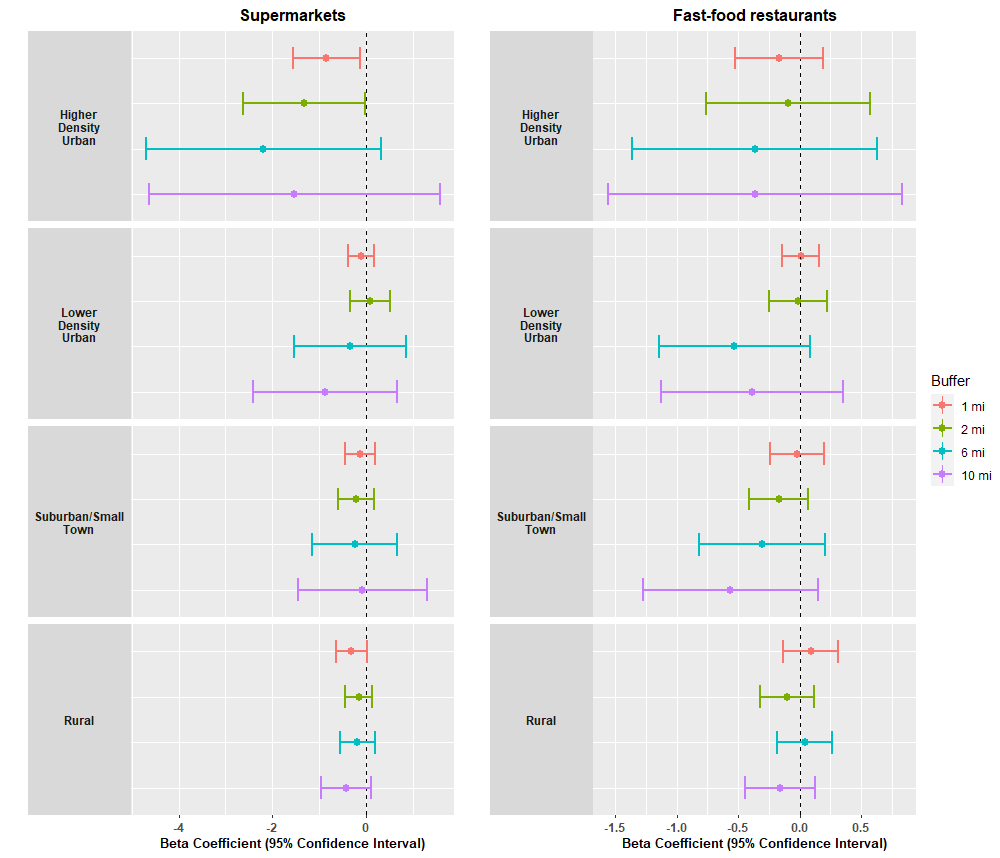


NOTE. Figure shows increase in Mediterranean diet score per each 1-unit increase in supermarket / fast-food restaurant availability. Food access is defined as the relative availability of two food outlets: the percentage of supermarkets out of all food stores, and the percentage of fast-food restaurants out of all restaurants.
