# Supplementary material for: Assessing the association between food environment and dietary inflammation by community type: a cross-sectional REGARDS study
Source: Int J Health Geogr. 2023 Sep 20;22:24. doi: 10.1186/s12942-023-00345-4 (PMC10510199; doi:10.1186/s12942-023-00345-4)
Supplement: Supplementary file 2 — Additional file 2: Table S1. Unadjusted sociodemographic characteristics of REGARDS participants excluded due to missing data. [file 12942_2023_345_MOESM2_ESM.docx]

| **SUPPLEMENTARY TABLE 1. Unadjusted sociodemographic characteristics of REGARDS participants excluded due to missing data** | |
| --- | --- |
| Characteristic | Excluded (n=9861)^a^ |
| Age, mean (SD) | 64.89 (9.84) |
| Sex, n (%) |  |
| Male | 4574 (46.39) |
| Female | 5287 (53.62) |
| Education, n (%) |  |
| Less than high school | 1869 (18.95) |
| High school graduate | 2646 (26.83) |
| Some college | 2519 (25.55) |
| College graduate and above | 2802 (28.41) |
| Missing | 25 (0.25) |
| Race, n (%) |  |
| Black | 5693 (57.73) |
| White | 4168 (42.27) |
| Income, n (%) |  |
| <$20000 | 2319 (23.52) |
| $20000-$34000 | 2398 (24.32) |
| $35000-$74000 | 2508 (25.43) |
| $75000 and above | 1221 (12.38) |
| Refused | 1415 (14.35) |
| ^a^Participants excluded due to missing data include those missing DIS, census tract identifier or sociodemographic data. | |
