# Supplementary material for: Assessing the association between food environment and dietary inflammation by community type: a cross-sectional REGARDS study
Source: Int J Health Geogr. 2023 Sep 20;22:24. doi: 10.1186/s12942-023-00345-4 (PMC10510199; doi:10.1186/s12942-023-00345-4)
Supplement: Supplementary file 3 — Additional file 3: Table S2. Cross-tabulations of dietary scores across individuals living in higher density urban census tracts (n=3389). [file 12942_2023_345_MOESM3_ESM.docx]

| **SUPPLEMENTARY TABLE 2. Cross-tabulations of dietary scores across individuals living in higher density urban census tracts (n=3389)** | | | |
| --- | --- | --- | --- |
|  | Mediterranean Diet Score Tertile 1, n (%) | Mediterranean Diet Score Tertile 2, n (%) | Mediterranean Diet Score Tertile 3, n (%) |
| DIS Tertile 1, n (%) | 143 (13.62) | 395 (37.62) | 512 (48.76) |
| DIS Tertile 2, n (%) | 249 (23.58) | 491 (46.50) | 316 (29.92) |
| DIS Tertile 3, n (%) | 612 (47.70) | 503 (39.20) | 168 (13.09) |
| Note. The highest tertile reflects a more pro-inflammatory diet and higher adherence to Mediterranean diet for DIS and Mediterranean Diet Score, respectively. | | | |
