# Supplementary material for: Assessing the association between food environment and dietary inflammation by community type: a cross-sectional REGARDS study
Source: Int J Health Geogr. 2023 Sep 20;22:24. doi: 10.1186/s12942-023-00345-4 (PMC10510199; doi:10.1186/s12942-023-00345-4)
Supplement: Supplementary file 4 — Additional file 4: Table S3. Model-based associations of the food environment with Mediterranean diet score (n=20,322). [file 12942_2023_345_MOESM4_ESM.docx]

| **SUPPLEMENTARY TABLE 3. Model-based associations of the food environment with Mediterranean diet score (n=20322)** | | |
| --- | --- | --- |
|  | Main effects model | |
|  | Β (SE) | p-value |
| Supermarkets, tailored^a^ | -0.26 (0.15) | 0.08 |
| Fast-food restaurants, tailored^a^ | 0.10 (0.08) | 0.22 |
| Community type |  |  |
| Higher density urban | 0.08 (0.05) | 0.10 |
| Lower density urban | **0.06 (0.03)** | **0.04** |
| Suburban/small town | 0.04 (0.03) | 0.25 |
| Rural (reference) |  |  |
| NOTE. Bold denotes statistically significant at α <0.05 level. Supermarkets and fast-food restaurants were modeled together. We controlled for individual-level covariates, NSEE, and total food outlets. Higher scores indicate greater adherence to a Mediterranean diet (theoretical range: 0–9). | | |
| ^a^We tailored buffer sizes to each community type using 2-, 3-, 10-, and 16-km (1-, 2-, 6-, and 10-mile) buffers for higher density urban, lower density urban, suburban/small town, rural areas, respectively. Buffer sizes are represented in kilometers rounded to the nearest whole number. | | |
