# Supplementary material for: Assessing the association between food environment and dietary inflammation by community type: a cross-sectional REGARDS study
Source: Int J Health Geogr. 2023 Sep 20;22:24. doi: 10.1186/s12942-023-00345-4 (PMC10510199; doi:10.1186/s12942-023-00345-4)
Supplement: Supplementary file 5 — Additional file 5: Table S4. Model-based associations of the food environment with Mediterranean diet score stratified by community type (n=20,322). [file 12942_2023_345_MOESM5_ESM.docx]

| **Additional file 5: Table 4. Model-based associations of the food environment with Mediterranean diet score stratified by community type (n=20,322)** | | | | | | | | |
| --- | --- | --- | --- | --- | --- | --- | --- | --- |
|  | Higher Density Urban | | Lower Density Urban | | Suburban/Small town | | Rural | |
|  | β (SE) | p-value | β (SE) | p-value | β (SE) | p-value | β (SE) | p-value |
| *Supermarkets* |  |  |  |  |  |  |  |  |
| Percentage, tailored^a^ | -0.84 (0.36) | 0.02 | 0.09 (0.22) | 0.69 | -0.24 (0.47) | 0.61 | -0.42 (0.27) | 0.11 |
|  |  |  |  |  |  |  |  |  |
| *Fast-food restaurants* |  |  |  |  |  |  |  |  |
| Percentage, tailored^a^ | -0.16 (0.18) | 0.37 | -0.01 (0.12) | 0.91 | -0.30 (0.26) | 0.24 | -0.16 (0.15) | 0.27 |
| NOTE. Signiﬁcance tests of our subgroup analyses employed a Bonferroni-corrected alpha level of *P* ≤ 0.01. Supermarkets and fast-food restaurants were modeled together. We controlled for individual-level covariates, NSEE, and total food outlets. Higher scores indicate greater adherence to a Mediterranean diet (theoretical range: 0–9). | | | | | | | | |
| ^a^We tailored buffer sizes to each community type using 2-, 3-, 10-, and 16-km (1-, 2-, 6-, and 10-mile) buffers for higher density urban, lower density urban, suburban/small town, and rural areas, respectively. Buffer sizes are represented in kilometers rounded to the nearest whole number. | | | | | | | | |
