# Supplementary material for: Assessing the association between food environment and dietary inflammation by community type: a cross-sectional REGARDS study
Source: Int J Health Geogr. 2023 Sep 20;22:24. doi: 10.1186/s12942-023-00345-4 (PMC10510199; doi:10.1186/s12942-023-00345-4)
Supplement: Supplementary file 6 — Additional file 6: Table S5. Relative measures of the food environment by community type and buffer size. [file 12942_2023_345_MOESM6_ESM.docx]

| **Additional file 6: Table 5. Relative measures of the food environment by community type and buffer size** | | | | |
| --- | --- | --- | --- | --- |
|  | Higher density urban | Lower density urban | Suburban/Small town | Rural |
| Supermarkets, mean (SD) | |  |  |  |
| Percentage, 2 km | - | 0.08 (0.13) | 0.07 (0.15) | 0.05 (0.14) |
| Percentage, 3 km | 0.10 (0.04) | - | 0.11 (0.13) | 0.09 (0.16) |
| Percentage, 10 km | 0.10 (0.02) | 0.11 (0.03) | - | 0.12 (0.11) |
| Percentage, 16 km | 0.10 (0.02) | 0.11 (0.02) | 0.12 (0.03) | - |
| Fast food restaurants, mean (SD) | | |  |  |
| Percentage, 2 km | - | 0.26 (0.23) | 0.18 (0.26) | 0.08 (0.21) |
| Percentage, 3 km | 0.29 (0.10) | - | 0.27 (0.22) | 0.22 (0.24) |
| Percentage, 10 km | 0.27 (0.07) | 0.33 (0.07) | - | 0.27 (0.22) |
| Percentage, 16 km | 0.26 (0.07) | 0.32 (0.06) | 0.34 (0.07) | - |
| Total food outlets, mean (SD) | |  |  |  |
| Density, 2 km | - | 4.74 (4.10) | 2.56 (3.16) | 1.46 (2.35) |
| Density, 3 km | 13.94 (17.98) | - | 2.50 (2.21) | 1.27 (1.59) |
| Density, 10 km | 12.29 (14.73) | 4.11 (2.79) | - | 0.48 (0.48) |
| Density, 16 km | 10.59 (12.32) | 3.43 (2.94) | 1.28 (1.10) | - |
| NOTE. Buffer sizes are represented in kilometers rounded to the nearest whole number. Primary buffer sizes (2 km / 1 mi for higher density urban, 3 km / 2 mi for lower density urban, 10 km / 6 mi for suburban/small town, and 16 km / 10 mi for rural) were presented in Table 2 and omitted from this table. All food stores and all restaurants were used as the denominators for the percentage of supermarkets and fast-food restaurants, respectively. Percentages have been converted to decimals. | | | | |
