# Supplementary material for: Assessing the association between food environment and dietary inflammation by community type: a cross-sectional REGARDS study
Source: Int J Health Geogr. 2023 Sep 20;22:24. doi: 10.1186/s12942-023-00345-4 (PMC10510199; doi:10.1186/s12942-023-00345-4)
Supplement: Supplementary file 7 — Additional file 7: Table S6. Model-based associations of the food environment with DIS by buffer size and community type. [file 12942_2023_345_MOESM7_ESM.docx]

| **Additional file 7: Table S6. Model-based associations of the food environment with DIS by buffer size and community type** | | | | | | | | |
| --- | --- | --- | --- | --- | --- | --- | --- | --- |
|  | Higher Density Urban | | Lower Density Urban | | Suburban/Small town | | Rural | |
|  | β (SE) | p-value | β (SE) | p-value | β (SE) | p-value | β (SE) | p-value |
| *Supermarkets* |  |  |  |  |  |  |  |  |
| Percentage, 2 km | **-** | **-** | -0.07 (0.20) | 0.72 | -0.01 (0.22) | 0.97 | 0.17 (0.22) | 0.45 |
| Percentage, 3 km | 1.61 (0.92) | 0.08 | **-** | **-** | 0.08 (0.29) | 0.78 | 0.17 (0.19) | 0.38 |
| Percentage, 10 km | 2.63 (1.89) | 0.16 | 1.43 (0.88) | 0.10 | **-** | **-** | 0.07 (0.30) | 0.82 |
| Percentage, 16 km | 2.44 (2.39) | 0.31 | 1.51 (1.14) | 0.19 | -0.28 (1.07) | 0.79 | **-** | **-** |
| *Fast-food restaurants* | |  |  |  |  |  |  |  |
| Percentage, 2 km | **-** | **-** | 0.17 (0.11) | 0.11 | **0.39 (0.14)** | **<.01** | -0.08 (0.15) | 0.60 |
| Percentage, 3 km | **1.26 (0.51)** | **0.01** | **-** | **-** | **0.46 (0.17)** | **<.01** | 0.04 (0.15) | 0.80 |
| Percentage, 10 km | **2.25 (0.78)** | **<.01** | **1.64 (0.45)** | **<.001** | **-** | **-** | 0.03 (0.16) | 0.88 |
| Percentage, 16 km | 1.99 (0.90) | 0.03 | **1.80 (0.54)** | **<.001** | **2.19 (0.55)** | **<.001** | **-** | **-** |
| NOTE. n=20322. Bold denotes statistically significant at Bonferroni-corrected α <0.01 level. Supermarkets and fast-food restaurants were modeled together. We controlled for individual-level covariates, NSEE, and total food outlets. Higher scores indicate more proinflammatory diets (theoretical range: -14.9–12.8). Buffer sizes are represented in kilometers rounded to the nearest whole number. Primary buffer sizes (2 km / 1 mi for higher density urban, 3km / 2 mi for lower density urban, 10 km / 6 mi for suburban/small town, and 16 km / 10 mi for rural) were presented in Table 2 and omitted from this table. | | | | | | | | |
