# Supplementary material for: Assessing the association between food environment and dietary inflammation by community type: a cross-sectional REGARDS study
Source: Int J Health Geogr. 2023 Sep 20;22:24. doi: 10.1186/s12942-023-00345-4 (PMC10510199; doi:10.1186/s12942-023-00345-4)
Supplement: Supplementary file 8 — Additional file 8: Table S7. Model-based associations of the food environment with Mediterranean diet score by community type and buffer size. [file 12942_2023_345_MOESM8_ESM.docx]

| **Additional file 8: Table S7. Model-based associations of the food environment with Mediterranean diet score** **by community type and buffer size** | | | | | | | | |
| --- | --- | --- | --- | --- | --- | --- | --- | --- |
|  | Higher Density Urban | | Lower Density Urban | | Suburban/Small town | | Rural | |
|  | β (SE) | p-value | β (SE) | p-value | β (SE) | p-value | β (SE) | p-value |
| *Supermarkets* |  |  |  |  |  |  |  |  |
| Percentage, 1 mi | **-** | **-** | -0.10 (0.14) | 0.49 | -0.13 (0.16) | 0.42 | -0.31 (0.17) | 0.07 |
| Percentage, 2 mi | -1.32 (0.66) | 0.05 | **-** | **-** | -0.20 (0.19) | 0.29 | -0.15 (0.15) | 0.32 |
| Percentage, 6 mi | -2.19 (1.28) | 0.09 | -0.34 (0.61) | 0.57 | **-** | **-** | -0.18 (0.19) | 0.35 |
| Percentage, 10 mi | -1.53 (1.59) | 0.33 | -0.87 (0.78) | 0.26 | -0.08 (0.70) | 0.91 | **-** | **-** |
| *Fast-food restaurants* | |  |  |  |  |  |  |  |
| Percentage, 1 mi | **-** | **-** | 0.01 (0.08) | 0.91 | -0.02 (0.11) | 0.85 | 0.09 (0.11) | 0.43 |
| Percentage, 2 mi | -0.09 (0.34) | 0.78 | **-** | **-** | -0.17 (0.12) | 0.16 | -0.10 (0.11) | 0.37 |
| Percentage, 6 mi | -0.36 (0.51) | 0.47 | -0.53 (0.31) | 0.09 | **-** | **-** | 0.04 (0.11) | 0.72 |
| Percentage, 10 mi | -0.37 (0.61) | 0.55 | -0.39 (0.38) | 0.31 | -0.56 (0.37) | 0.12 | **-** | **-** |
| NOTE. n=20322. Signiﬁcance tests of our subgroup analyses employed a Bonferroni-corrected alpha level of *P* ≤ 0.01. Supermarkets and fast-food restaurants were modeled together. We controlled for individual-level covariates, NSEE, and total food outlets. Higher scores indicate greater adherence to a Mediterranean diet (theoretical range: 0–9). Primary buffer sizes (1 mi for higher density urban, 2 mi for lower density urban, 6 mi for suburban/small town, and 10 mi for rural) were presented in Table 2 and omitted from this table. | | | | | | | | |
